# Graphene Far-Infrared Therapy Enhances Diabetic Wound Healing Through Potential Mitigation of Oxidative Stress and Inflammation and Regulation of Chemokines and Macrophage Polarization

**DOI:** 10.3390/ijms27073101

**Published:** 2026-03-29

**Authors:** Xinyu Jian, Xuanjun Wu, Xian Luo, Chengwei Cao, Qianwen Wu, Ziwen Chen, Zhichao Hu, Hua Zhu, Binghui Wu

**Affiliations:** Innovation Laboratory for Sciences and Technologies of Energy Materials of Fujian Province (IKKEM), State Key Laboratory for Physical Chemistry of Solid Surfaces, Collaborative Innovation Center of Chemistry for Energy Materials (iChEM), National and Local Joint Engineering Research Center of Preparation Technology of Nanomaterials, College of Chemistry and Chemical Engineering, Pen-Tung Sah Institute of Micro-Nano Science and Technology, College of Medicine, Xiamen University, Xiamen 361005, China; jianxinyu2001@stu.xmu.edu.cn (X.J.); 36520211151875@stu.xmu.edu.cn (X.W.); 24520220157307@stu.xmu.edu.cn (X.L.); 17720880695@163.com (C.C.); 36520221151880@stu.xmu.edu.cn (Q.W.); 36520211151718@stu.xmu.edu.cn (Z.C.); hu13545590469@163.com (Z.H.); zh_980903@163.com (H.Z.)

**Keywords:** diabetic wound healing, far-infrared radiation (FIR) therapy, immunomodulation, reactive oxygen species, angiogenesis, NF-κB signaling

## Abstract

Chronic diabetic wounds remain difficult to heal because persistent inflammation, oxidative stress, and impaired regeneration delay repair, while effective noninvasive options are limited. In this study, graphene-based far-infrared radiation (FIR) therapy was evaluated in a streptozotocin (STZ)-induced diabetic rat full-thickness wound model, and mechanisms were examined in vivo and in vitro. Wound closure was quantified by serial imaging, whereas tissue remodeling and angiogenesis were assessed by H&E and Masson’s trichrome staining and CD34-based analyses. Transcriptomic responses were profiled by RNA sequencing with qRT-PCR validation, immune phenotypes were characterized by immunofluorescence, and high-glucose cell assays were performed. Re-epithelialization, collagen deposition, and neovascularization were quantified histologically. These datasets enabled integrated evaluation of inflammation, oxidative stress, and repair programs over time. Graphene FIR accelerated closure, reaching 83.9% healing by day 14 vs. 66.8% in untreated controls. Treatment was associated with downregulation of Cxcl2/Cxcl3, suppression of M1 polarization with enhanced M2 polarization, and reduced ROS accumulation. Consistently, NF-κB signaling was inhibited, supporting restoration of a pro-regenerative microenvironment. Collectively, graphene FIR represents a promising noninvasive strategy for diabetic wound repair via coordinated immunomodulatory and antioxidant actions.

## 1. Introduction

Diabetes mellitus (DM) is a chronic metabolic disorder characterized by insufficient insulin secretion or impaired insulin sensitivity, leading to disrupted glucose, lipid, and protein metabolism [1,2]. According to the 2021 International Diabetes Federation, approximately 537 million adults are affected globally, with this number projected to surpass 700 million by 2045 [3]. Persistent hyperglycemia in diabetic individuals contributes to progressive tissue and organ damage, resulting in serious complications such as diabetic foot ulcers (DFUs)—a leading cause of amputation and disability worldwide [4,5,6,7,8]. Clinically, DFUs are particularly difficult to treat due to the combined effects of vascular impairment, peripheral neuropathy, and immune dysfunction [9,10,11,12]. These pathologies converge to disrupt normal wound healing, leading to prolonged inflammation, impaired angiogenesis, and delayed tissue regeneration [13,14]. The failure to resolve inflammation effectively remains one of the central challenges in diabetic wound healing.

Normal wound healing proceeds through overlapping stages: hemostasis, inflammation, proliferation, and remodeling [15]. In diabetes, this orderly process is disrupted, particularly at the inflammatory phase. Excessive and sustained production of reactive oxygen species (ROS) in hyperglycemic conditions damages fibroblasts, impairs endothelial function, and intensifies inflammatory signaling [16,17]. This environment promotes persistent neutrophil infiltration and M1 macrophage polarization, both of which contribute to prolonged cytokine release, notably Tnf-α and IL-1β [18,19]. These factors collectively hinder the transition to the proliferative phase, disrupting fibroblast proliferation and keratinocyte migration. As a result, diabetic wounds often fail to progress beyond inflammation, culminating in chronic non-healing ulcers.

Numerous therapeutic interventions have been developed to enhance diabetic wound healing, including pharmacologic agents, surgical debridement, hyperbaric oxygen therapy, and growth factor or stem cell-based approaches [20,21,22,23,24]. However, these modalities are often limited by high cost, invasive procedures, or inconsistent clinical efficacy. Recently, far-infrared radiation (FIR) therapy has attracted growing interest as a non-invasive adjunct to wound management [25,26,27]. FIR has been shown to improve local circulation, enhance fibroblast viability, and modulate inflammatory signaling, making it suitable for promoting tissue regeneration [28]. Yet, the application of conventional FIR devices is constrained by low thermal efficiency, limited tissue penetration, and poor stability, limiting their translational utility in clinical practice.

To overcome these limitations, graphene-based materials have emerged as next-generation FIR emitters due to their superior thermal conductivity, high emissivity, and mechanical flexibility [29]. Graphene-based FIR platforms can convert external energy sources into stable far-infrared output, offering improved precision and efficiency compared to metal-based FIR systems. Their tunable heating properties and biocompatibility make them particularly suitable for chronic wound applications [30]. In previous studies, graphene FIR has been demonstrated to significantly enhance T-cell immunocompetence and suppress melanoma progression [31,32]. The primary objective of this study was to systematically evaluate the therapeutic efficacy of graphene-based FIR treatment in diabetic wound healing and to elucidate the underlying mechanisms. The novelty of this work lies in a comprehensive assessment of the multi-faceted effects of graphene FIR—encompassing regulation of inflammatory chemokines, macrophage polarization, and oxidative stress—which have not been extensively characterized in the context of diabetic wound repair. In this study, we developed a graphene FIR device and systematically evaluated its efficacy in promoting diabetic wound healing. Specifically, we investigated its ability to modulate inflammatory chemokines (Cxcl2, Cxcl3), regulate macrophage polarization, reduce neutrophil infiltration, and attenuate ROS accumulation. By targeting key pathways involved in immune cell recruitment, oxidative stress, and cytokine secretion, graphene FIR offers a promising, non-invasive therapeutic strategy to address the persistent challenges of diabetic wound care.

## 2. Results

### 2.1. Therapeutic Effects of Graphene FIR in Diabetic Wound Healing

Emission spectral analysis revealed that both the graphene film (GF) and the metal film (MF) exhibited distinct emission peaks within 10–16 μm, a range commonly regarded as the FIR biological window. The GF-FIR device demonstrated high thermal stability and strong FIR emissivity, with an electrical-to-thermal conversion efficiency of 68%. In contrast, the MF device, which was used as a low-intensity FIR control, showed a lower conversion efficiency of 52%.

**Figure 1:**
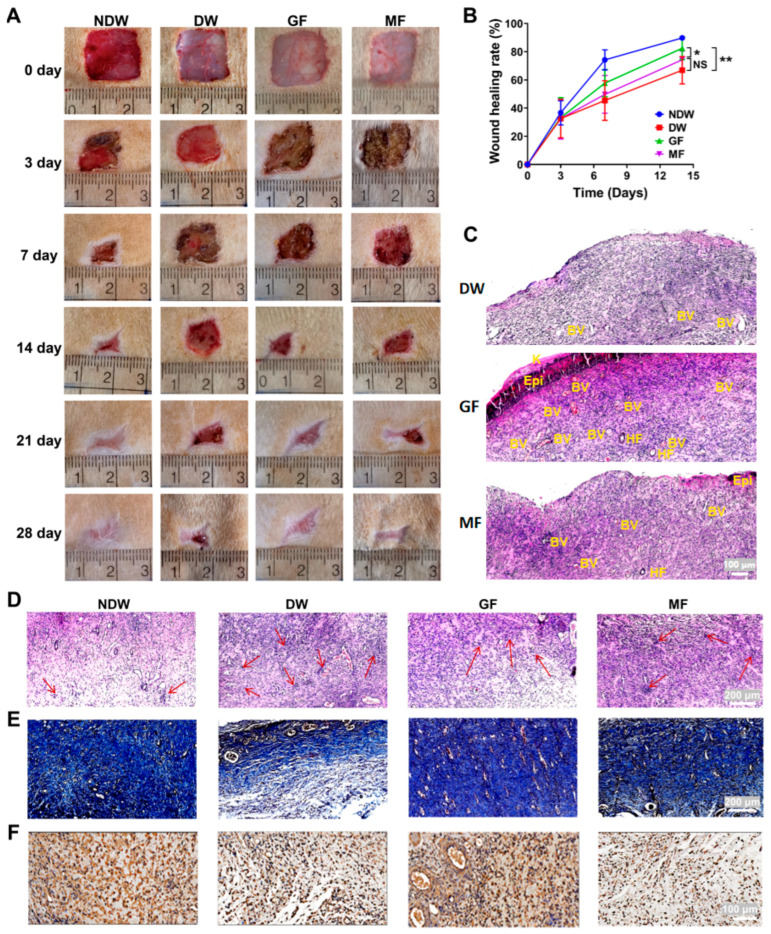
Therapeutic effects of graphene FIR in diabetic wound healing. (**A**) Representative images showing wound morphology on days 0, 3, 7, 14, 21, and 28 post-treatment across different groups. (**B**) Quantitative analysis of wound closure rates as a function of days. (**C**) Microscopic images of wound tissues at 7 days post-treatment. Epi: epidermis; K: keratin layer; BV: blood vessels; HF: hair follicles. (**D**–**F**) HE staining, Masson’s trichrome staining, and CD34 immunohistochemical staining of skin tissue at 7 days post-treatment. Statistical significance is indicated as NS *p* > 0.05, * *p* < 0.05, ** *p* < 0.01. The red arrow indicates an inflammatory cell.

A localized treatment strategy was employed to evaluate the efficacy of graphene-based FIR therapy (Figure S1) [31,32] on diabetic wound healing. Wound morphology was recorded at days 0, 3, 7, 14, 21, and 28 post-treatment (Figure 1A), and closure rates were analyzed on days 7 and 14 (Figure 1B). By day 7, notable differences in healing outcomes had emerged among the groups. The non-diabetic wound (NDW) group showed the most advanced healing, with a 74.2% closure rate and visible epidermal remodeling, indicating entry into the remodeling phase. In contrast, the diabetic wound (DW) group exhibited no scab formation and presented with redness and swelling, indicative of prolonged inflammation. Its closure rate was only 45.3%, suggesting delayed wound resolution. In the GF and MF treatment groups, scab formation had occurred, and wound closure rates reached 56.5% and 49.7%, respectively. By day 7 post-treatment, wounds treated with GF/MF began to exhibit a thin layer of nascent epithelium accompanied by marked fibroblast proliferation (Figure 1C). Notably, GF-treated wounds showed more advanced skin regeneration, characterized by the reformation of the epidermis, organized keratinocyte layers, and emerging hair follicles, together with a higher density of newly formed blood vessels. These histological features indicate robust proliferation of both fibroblasts and keratinocytes, which likely supported epidermal restoration and promoted connective tissue formation and collagen deposition within the dermis. Collectively, these results suggest that GF treatment facilitated a more rapid resolution of inflammation and exerted a stronger pro-regenerative effect than MF.

By day 14, the NDW group wounds had nearly completed re-epithelialization, with an 89.8% closure rate. The DW group still exhibited signs of active inflammation, with unresolved dermal repair and a closure rate of 66.8%. In contrast, wounds in both the GF and MF groups had progressed into the remodeling phase. The GF group achieved an 83.9% closure rate, closely approximating that of the NDW group, while the MF group reached 74.2%, indicating intermediate healing. These findings support the conclusion that GF treatment accelerates wound closure more effectively than MF treatment, particularly under hyperglycemic conditions. Importantly, the nearly comparable healing outcomes between the GF and NDW groups suggest that graphene FIR can partially restore impaired healing capacity in diabetic wounds. This therapeutic advantage likely results from graphene FIR’s ability to target underlying pathological mechanisms associated with inflammation and tissue regeneration. Thus, in both early and later healing stages, GF outperformed MF, providing consistent improvements in wound resolution.

To explore tissue-level effects, HE staining, Masson’s trichrome staining, and CD34 immunohistochemistry were performed on wound samples collected at day 7. For each wound, representative tissue sections were evaluated in two non-overlapping high-power fields per section. Neutrophils and lymphocytes were manually counted in each high-power field. Epidermal thickness was quantified as the vertical distance from the basal layer to the stratum corneum at a minimum of five randomly selected sites per section using ImageJ 1.8.0 software.

HE staining revealed minimal inflammatory cell infiltration in the NDW group, whereas the DW group showed extensive leukocyte accumulation, confirming that inflammation remained unresolved. Both GF and MF treatments significantly reduced inflammatory infiltration compared with DW, with the GF group showing the most pronounced reduction. This result aligns with the observed differences in wound morphology and supports the role of GF in modulating the inflammatory microenvironment (Figure 1D). Furthermore, tissue structure analysis indicated superior healing in the NDW and GF groups. By day 7 post-treatment, the NDW, GF, and MF groups displayed abundant, dense, and well-organized collagen bundles, consistent with more mature type I collagen deposition and advanced tissue remodeling. In contrast, the DW group exhibited markedly sparse and disorganized collagen, appearing as a faint, delicate network suggestive of immature type III collagen with limited maturation. Although collagen deposition was moderately improved in the MF group, the highly organized architecture observed in the GF group was not achieved (Figure 1E).

A continuous epidermal layer (highlighted in Figure S3) was observed in both GF and MF groups, with significantly greater epidermal thickness in GF-treated wounds than in DW and MF. These findings suggest that GF treatment promotes earlier transition to the proliferative phase and faster epidermal restoration, possibly by suppressing inflammation and promoting keratinocyte activity.

Masson’s trichrome staining further supported the regenerative advantage of GF therapy. Collagen fiber deposition in the GF group was both extensive and well-organized, closely resembling that in the NDW group and exceeding levels observed in the DW and MF groups (Figure 1E). In contrast, collagen in the DW group appeared sparse and disorganized, indicating impaired matrix synthesis. The MF group showed moderate collagen deposition, but the density and organization remained inferior to GF. Since collagen is critical for dermal integrity, fibroblast function, and mechanical support, its early and structured formation in the GF group suggests an accelerated transition into the remodeling phase. This structural maturity implies that graphene FIR not only enhances inflammatory resolution but also actively supports fibroblast-mediated tissue repair. The data highlight that graphene FIR promotes robust matrix regeneration, providing a solid architectural foundation essential for long-term wound stability and re-epithelialization. These outcomes confirm that GF treatment improves both the speed and quality of tissue repair.

To evaluate angiogenesis, CD34 immunohistochemical staining was used to assess vascular regeneration, as CD34 is a recognized marker of endothelial cells [33]. The NDW group exhibited dense, evenly distributed CD34-positive staining, indicating active microvessel formation. In contrast, the DW group showed markedly reduced CD34 staining, suggesting that hyperglycemia severely impairs neovascularization. Importantly, the GF group exhibited CD34 expression comparable to the NDW group, with clearly visible vascular structures throughout the dermis (Figure 1F). This suggests that graphene FIR significantly promotes angiogenesis, which is essential for supplying oxygen and nutrients to regenerating tissues. The MF group demonstrated moderate improvement in CD34 positivity relative to DW but still lagged behind GF. These results underscore the superior ability of graphene FIR to restore vascular function in diabetic wounds. Enhanced angiogenesis likely contributes to the improved wound closure, collagen deposition, and epidermal regeneration observed in the GF group, highlighting the multifactorial therapeutic effects of graphene FIR in the context of diabetic tissue repair.

### 2.2. Anti-Inflammatory Effects Through Regulation of Cxcl2 and Cxcl3

**Figure 2:**
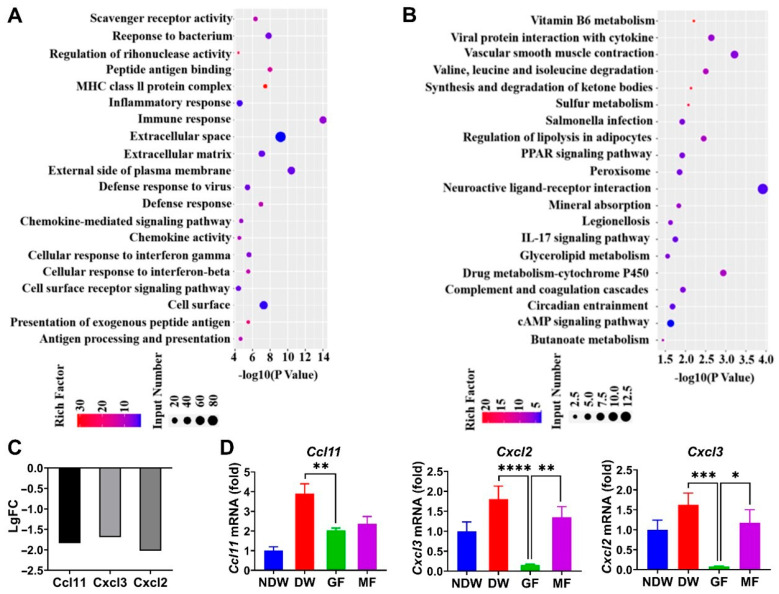
Bulk RNA-Seq analysis and mRNA expression levels of chemokines in skin tissue. (**A**) GO enrichment analysis of differentially expressed genes (DEGs) in the GF_DW group at day 7 post-treatment. (**B**) KEGG pathway enrichment analysis of DEGs in the GF_DW group. (**C**) Expression levels of co-enriched genes involved in the “Inflammatory response” and “IL-17 signaling pathway” categories. (**D**) Relative mRNA expression levels of *CCL11*, *Cxcl2*, and *Cxcl3* (n = 3). Data are presented as mean ± SEM from three independent experiments. Statistical significance is indicated as * *p* < 0.05, ** *p* < 0.01, *** *p* < 0.001, and **** *p* < 0.0001.

Following the confirmation of graphene FIR’s therapeutic efficacy in promoting diabetic wound healing, we investigated its underlying anti-inflammatory mechanisms. Bulk RNA sequencing (RNA-Seq) was conducted on wound tissues from the DW, GF, and MF groups at day 7 post-treatment to identify potential molecular drivers of healing. Differential gene expression analysis revealed 314 significantly upregulated and 212 downregulated genes in the GF group compared to DW (Figure S4). These data indicate that graphene FIR exerts substantial regulatory effects at the transcriptomic level. To better understand these regulatory effects, Gene Ontology (GO) and Kyoto Encyclopedia of Genes and Genomes (KEGG) enrichment analyses were conducted. As shown in Figure 2A,B, the GO analysis revealed significant enrichment of the “inflammatory response” category, while KEGG analysis identified the “IL-17 signaling pathway” as one of the most significantly enriched pathways. IL-17 signaling is known to drive chronic inflammation through activation of NF-κB and JAK-STAT pathways, upregulation of pro-inflammatory cytokines, and stimulation of chemokine secretion, thereby promoting leukocyte infiltration and prolonging inflammatory responses [34,35,36].

To explore this further, we analyzed co-enriched genes associated with both “inflammatory response” and “IL-17 signaling” terms. Among the most prominently enriched genes were chemokines Cxcl2, Cxcl3, and CCL11, all located downstream of IL-17 signaling (Figure 2C). These chemokines are pivotal regulators of immune cell recruitment and activation, especially neutrophils and M1 macrophages, which are central to the pathogenesis of chronic inflammation in diabetic wounds. To validate the transcriptomic findings, quantitative real-time PCR (qRT-PCR) was performed to assess the mRNA levels of these chemokines in wound tissues (Figure 2D). In the DW group, *Cxcl2* and *Cxcl3* expression was markedly elevated, consistent with sustained inflammatory signaling in hyperglycemic conditions. Notably, GF treatment significantly downregulated both *Cxcl2* and *Cxcl3* expression compared to DW, suggesting suppression of chemokine-mediated inflammatory cell recruitment. MF treatment also reduced their expression, but to a lesser extent than GF.

Interestingly, no significant difference in *CCL11* expression was observed between the GF and MF groups, suggesting that GF’s anti-inflammatory effects are more selectively targeted toward Cxcl2 and Cxcl3 regulation. These findings support the hypothesis that the suppression of these two chemokines represents a key mechanism by which graphene FIR mitigates inflammation in diabetic wounds. Given the role of Cxcl2 and Cxcl3 in neutrophil recruitment and M1 macrophage polarization, their downregulation likely contributes to a more favorable wound microenvironment conducive to repair. Collectively, these results indicate that graphene FIR exerts its therapeutic effects, at least in part, through modulation of IL-17-mediated chemokine expression (especially *Cxcl2* and *Cxcl3*), leading to reduced leukocyte infiltration and resolution of chronic inflammation.

### 2.3. Anti-Inflammatory Effects via Macrophage Polarization and Neutrophil Recruitment

**Figure 3:**
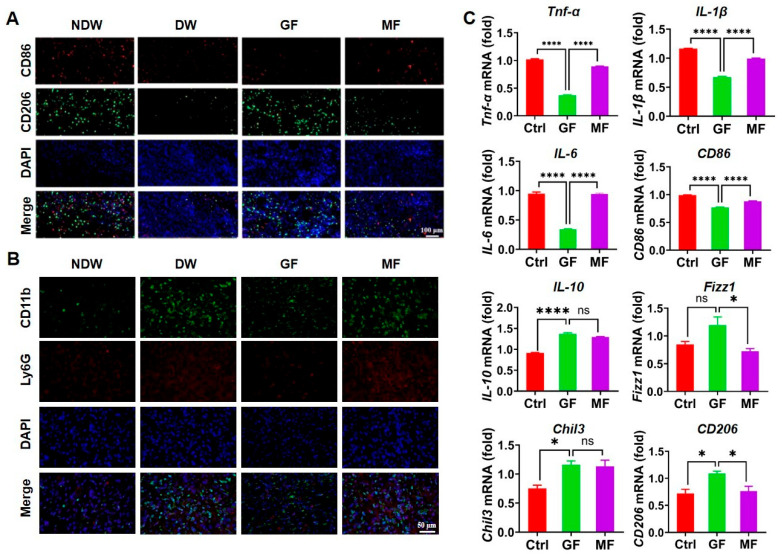
Graphene FIR alleviates inflammation by suppressing Cxcl2 and Cxcl3 expression. (**A**) Immunofluorescence staining of macrophages and (**B**) neutrophils in skin tissue at day 7 post-treatment. (**C**) Relative mRNA expression levels of anti-inflammatory cytokines, pro-inflammatory cytokines, and M1/M2 macrophage markers in RAW264.7 cells following graphene FIR treatment (n = 3). Data are presented as mean ± SEM from three independent experiments. Statistical significance is indicated as * *p* < 0.05, **** *p* < 0.0001, ns *p* > 0.05.

Previous qRT-PCR analyses demonstrated that graphene FIR significantly downregulated the expression of *Cxcl2* and *Cxcl3*—chemokines implicated in the recruitment of neutrophils and M1-polarized macrophages [37,38,39,40]. Elevated levels of these chemokines are associated with chronic inflammation, sustained M1 activation, and impaired development of reparative M2 macrophages, which are essential for tissue regeneration [41,42]. To examine the impact of graphene FIR on macrophage polarization, immunofluorescence staining was performed using CD86 as an M1 marker and CD206 as an M2 marker (Figure 3A). After 7 days of treatment, the DW group showed intense CD86 staining, indicating a dominance of M1 macrophages and a persistently inflammatory wound environment. In contrast, the GF group exhibited a marked reduction in CD86 signal, suggesting effective suppression of M1 polarization. The MF group also showed reduced CD86 intensity but to a lesser extent, indicating that conventional FIR has some modulatory effects, though not as robust as graphene FIR.

In parallel, analysis of CD206 staining revealed a significantly increased presence of M2 macrophages in the GF group compared to DW and MF groups. The DW group exhibited limited CD206 positivity, consistent with a deficient anti-inflammatory response. The MF group demonstrated moderate M2 expression, but not to the same degree as GF. These findings suggest that graphene FIR not only inhibits M1 polarization but also facilitates M2 macrophage differentiation, thereby reshaping the immune landscape toward a pro-repair phenotype. Consistent with this finding, immunofluorescence analysis revealed substantial infiltration of CD11b + Ly6G + neutrophils in the DW group, which was markedly reduced by graphene FIR (GF) treatment. In contrast, the metal-based FIR (MF) group showed only a modest, non-significant decrease in neutrophil infiltration (Figure 3B).

To further validate these findings, we conducted in vitro experiments using RAW264.7 macrophages. Cells were first polarized to the M1 phenotype using lipopolysaccharide (LPS) and interferon-γ (IFN-γ), followed by treatment with either GF or MFs. Untreated cells served as controls. qRT-PCR was used to quantify gene expression levels of M1- and M2-associated markers. M1 macrophages are defined by elevated expression of *Tnf-α*, *IL-1β*, and *IL-6*, as well as the surface marker CD86 [43,44]. Compared to control, GF treatment significantly downregulated all four markers, indicating a strong suppressive effect on M1 activity (Figure 3C). MF also reduced their expression, but to a lesser extent than GF, reinforcing the superior immunomodulatory effect of graphene FIR. These molecular findings corroborate the immunofluorescence data, confirming that GF effectively limits M1-driven inflammation at both transcriptional and phenotypic levels. Importantly, suppression of these pro-inflammatory mediators may contribute to the broader anti-inflammatory environment necessary for efficient wound healing.

To assess M2 macrophage activation, we evaluated the expression of *IL-10*, *Fizz1*, *Chil3*, and *CD206*—hallmarks of anti-inflammatory and tissue-reparative functions [45,46]. *IL-10* expression was significantly upregulated in the GF group compared to both MF and control. *Fizz1* expression was also elevated in GF relative to MF, although the difference from control was not significant. *Chil3* expression was significantly increased in the GF group compared to the control but not different from MF. Notably, *CD206* expression was markedly higher in GF-treated cells than in either of the other groups. These results suggest that graphene FIR not only suppresses inflammatory responses but also actively promotes the reparative phenotype of macrophages. The combined enhancement of IL-10 and *CD206* expression further indicates a transition toward tissue repair and inflammation resolution.

Together, these results demonstrate that graphene FIR exerts a dual regulatory effect on macrophage polarization—suppressing M1-mediated inflammation and enhancing M2-associated tissue repair. Through both in vivo immunostaining and in vitro gene expression profiling, we established that GF treatment effectively reprograms macrophage function, shifting the local immune environment from pro-inflammatory to pro-regenerative. This immunomodulatory capacity plays a central role in resolving inflammation and accelerating tissue recovery in diabetic wounds, distinguishing graphene FIR as a superior therapeutic strategy compared to conventional FIR.

### 2.4. Anti-Inflammatory Effects via Attenuation of Oxidative Stress in High-Glucose Conditions

Reactive oxygen species (ROS) are known to play a dual role in wound healing [47,48,49]. At physiological levels, ROS act as signaling molecules that regulate fibroblast proliferation, migration, and matrix production, all of which are essential for tissue repair [50,51,52]. However, under diabetic conditions characterized by chronic hyperglycemia, ROS production becomes pathologically elevated [53]. This overproduction damages fibroblast membranes, disrupts mitochondrial function, and compromises DNA integrity, leading to increased apoptosis and impaired regeneration [54,55,56]. Moreover, excessive ROS activates key inflammatory pathways, such as NF-κB, resulting in elevated pro-inflammatory cytokine expression and sustained immune activation [57,58,59]. This creates a vicious cycle that exacerbates tissue injury and delays wound resolution. Therefore, targeting oxidative stress is a strategic approach for restoring healing capacity in diabetic wounds.

**Figure 4:**
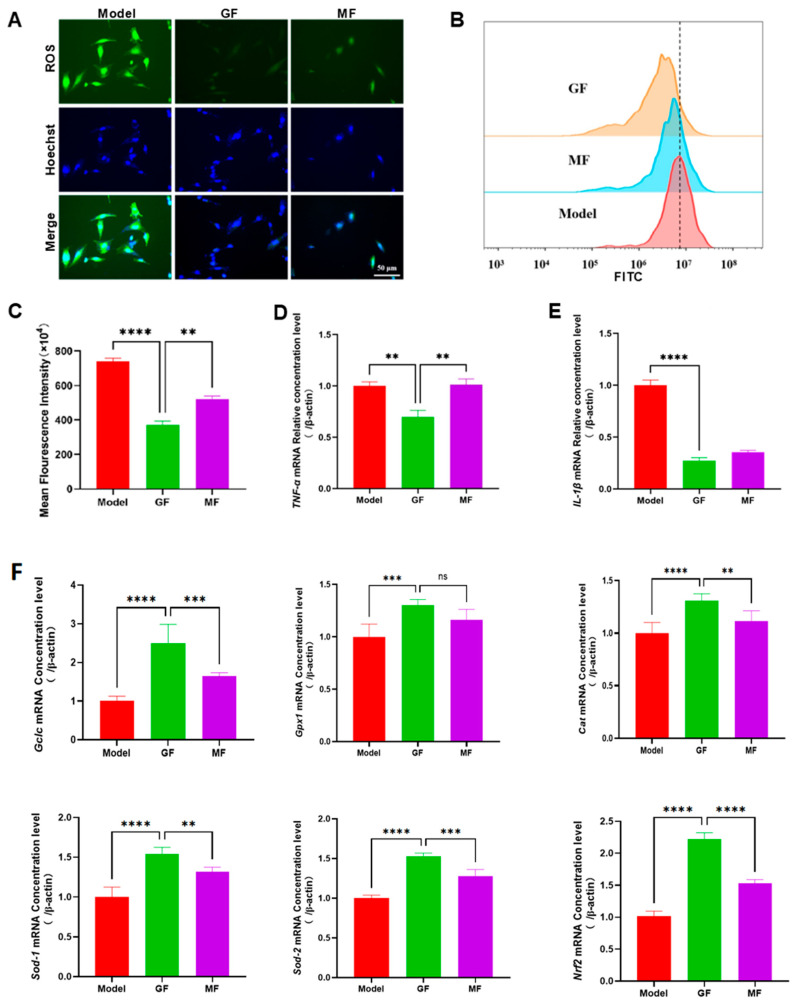
Graphene FIR inhibits inflammatory responses by reducing ROS production. (**A**) Immunofluorescence staining of intracellular ROS levels under different treatment conditions. (**B**) Flow cytometry histograms of ROS fluorescence intensity. (**C**) Quantitative analysis of intracellular ROS fluorescence intensity. (**D**,**E**) Relative mRNA expression levels of *Tnf-α* and *IL-1β* in HFF cells (n = 3). (**F**) Relative mRNA expression levels of oxidative stress-related biomarkers and their regulatory factor *Nrf2* in HFF cells (n = 3). Data are presented as mean ± SEM from three independent experiments. Statistical significance is indicated as ** *p* < 0.01, *** *p* < 0.001, and **** *p* < 0.0001, ns *p* > 0.05.

To investigate this mechanism, human foreskin fibroblasts (HFFs) were cultured in high-glucose (50 mmol/L) DMEM to simulate a diabetic microenvironment (Figure S5). ROS accumulation was assessed using the DCFH-DA fluorescent probe (Figure 4A). In the Model group, intense green fluorescence indicated excessive intracellular ROS. In contrast, the GF-treated group exhibited substantially weaker signals, suggesting effective suppression of ROS production. Although the MF group also showed reduced fluorescence, the intensity remained higher than that in the GF group, indicating inferior antioxidative efficacy. These qualitative observations suggest that graphene FIR more effectively mitigates high-glucose-induced oxidative stress compared to conventional FIR. This initial finding provides a mechanistic basis for its enhanced therapeutic performance in diabetic wound healing. To validate these results quantitatively, flow cytometry analysis was performed using the DCFH-DA probe (Figure 4B,C). In the Model group, a pronounced rightward shift in fluorescence intensity confirmed elevated ROS levels. The GF group demonstrated a marked leftward shift, indicating a significant reduction in ROS accumulation. The MF group also shifted leftward but to a lesser degree. Quantitative analysis of mean fluorescence intensity further confirmed these findings: GF treatment significantly reduced ROS levels compared to both the Model and MF groups. These results, consistent with immunofluorescence data, provide strong evidence that graphene FIR reduces oxidative stress more effectively than traditional FIR. Since ROS plays a central role in amplifying inflammation, its reduction is expected to suppress downstream cytokine expression and promote a regenerative environment.

To assess whether this ROS suppression was associated with reduced inflammatory signaling, we measured mRNA expression of *Tnf-α* and *IL-1β*—key pro-inflammatory cytokines activated by ROS-induced pathways [60,61]. In the Model group, expression levels of both cytokines were significantly elevated. In contrast, the GF group exhibited markedly reduced expression of *Tnf-α* and *IL-1β*. Therefore, the markedly reduced mRNA levels of *Tnf-α* and *IL-1β* in the GF group indicate that, in parallel with decreased ROS accumulation, graphene FIR may attenuate ROS-associated inflammatory signaling (Figure 4D,E). A partial reduction in these transcripts was also observed in the MF group, although the magnitude of change was smaller than that in the GF group. Overall, these data support an association between the antioxidant effects of graphene FIR and suppressed expression of pro-inflammatory cytokines, suggesting coordinated modulation of oxidative stress and inflammatory responses under hyperglycemic conditions. Notably, the involvement of specific downstream pathways (e.g., NF-κB) was not directly examined and should be validated in future studies.

To further assess activation of the Nrf2-dependent antioxidant program, the mRNA expression of canonical downstream enzymes (*Gclc*, *Gpx1*, *Cat*, *Sod-1*, *Sod-2* and *Nrf2*) was quantified. qRT-PCR analysis showed that these transcripts were significantly upregulated in both the GF and MF groups compared with the Model group (Figure 4F), consistent with enhanced antioxidant transcriptional activity. Moreover, the induction observed in the GF group was significantly greater than that in the MF group.

Taken together, graphene FIR reduced intracellular ROS levels in fibroblasts under high-glucose stress and was accompanied by decreased mRNA expression of pro-inflammatory cytokines (e.g., *Tnf-α* and *IL-1β*), supporting attenuation of ROS-related inflammatory responses. This dual effect disrupts the feedback loop between oxidative stress and inflammation, two core contributors to delayed wound healing in diabetes. Compared to conventional FIR, graphene FIR offers enhanced modulation of redox and immune balance, positioning it as a superior non-invasive intervention for restoring tissue repair capacity in oxidative microenvironments. These findings elucidate a critical mechanism by which graphene FIR contributes to inflammation resolution and regenerative restoration in diabetic wounds.

### 2.5. Anti-Inflammatory Effects via Suppression of Cytokine Expression in Diabetic Wounds

**Figure 5:**
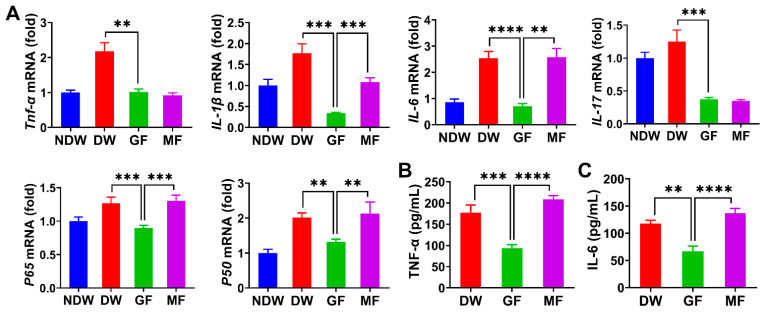
Changes in inflammatory markers in rat skin tissue after 7 days of treatment. (**A**) Relative mRNA expression levels of *Tnf-α*, *IL-1β*, *IL-6*, *IL-17*, *p65*, and *p50* in wound tissue (n = 3). (**B**,**C**) Protein expression levels of Tnf-α and IL-6 in wound tissue fluid, as measured by ELISA. Data are presented as mean ± SEM from three independent experiments. Statistical significance is indicated as ** *p* < 0.01, *** *p* < 0.001, and **** *p* < 0.0001.

Previous results demonstrated that graphene FIR suppressed Cxcl2 and Cxcl3 expression, reduced M1 macrophage polarization, enhanced M2 polarization, and inhibited ROS accumulation—mechanisms contributing to its anti-inflammatory effects. To validate these findings in vivo, we examined inflammatory cytokine expression in rat skin tissues using qRT-PCR (Figure 5A). In the diabetic wound (DW) group, mRNA levels of *Tnf-α*, *IL-1β*, *IL-6*, and *IL-17* were significantly elevated, consistent with increased M1 macrophages and neutrophil infiltration. In contrast, the GF group exhibited marked downregulation of these cytokines, particularly *IL-1β* and *IL-6*, indicating reduced inflammatory cell recruitment and cytokine release. While the MF group also showed reductions in *Tnf-α* and *IL-17*, levels of *IL-1β* and *IL-6* remained significantly higher than in the GF group. These results suggest that conventional FIR has limited anti-inflammatory efficacy compared to graphene FIR, which more effectively suppresses key pro-inflammatory cytokines under diabetic conditions.

As NF-κB is a key transcriptional regulator of inflammatory cytokines and chemokines such as Tnf-α, IL-1β, IL-6, IL-17, Cxcl2, and Cxcl3, we next evaluated its activation status [62,63]. qRT-PCR analysis showed significantly elevated mRNA levels of *p65* and *p50*—the primary components of the NF-κB complex—in the DW group, confirming pathway activation. This downregulation occurred in parallel with a reduction in pro-inflammatory cytokines known to be regulated by NF-κB. While this pattern is consistent with a modulation of the NF-κB signaling axis, further protein-level analyses (e.g., phosphorylation or nuclear translocation) are needed to confirm the direct impact on pathway activity. The MF group also exhibited a reduction, though less pronounced, aligning with its weaker suppression of cytokines. These results highlight that graphene FIR attenuates inflammation not only by targeting chemokine expression and oxidative stress but also by modulating key transcriptional pathways involved in chronic immune activation.

To assess whether transcriptional suppression was reflected at the protein level, ELISA was conducted to quantify Tnf-α and IL-6 concentrations in wound tissue fluids (Figure 5B,C). Tnf-α levels were 177 pg/mL in the DW group and 208 pg/mL in the MF group, whereas the GF group showed significantly lower levels at 94 pg/mL. Similarly, IL-6 levels in the DW and MF groups were 117 pg/mL and 137 pg/mL, respectively, compared to only 66 pg/mL in the GF group. These findings indicate that GF reduced Tnf-α and IL-6 protein levels by approximately 50% relative to both DW and MF. The greater suppression observed in the GF group supports its enhanced ability to regulate inflammatory mediators at multiple molecular levels.

Taken together, these data demonstrate that graphene FIR significantly suppresses pro-inflammatory cytokine expression at both transcriptional and protein levels in diabetic wounds. This suppression is likely mediated through multiple upstream mechanisms, including inhibition of NF-κB signaling and Cxcl2/Cxcl3-driven immune cell recruitment. By simultaneously modulating chemokine signaling, inflammatory cytokines, and transcription factor activity, graphene FIR creates a more favorable microenvironment for wound healing. These multi-tiered effects reinforce its potential as an advanced therapeutic modality for mitigating chronic inflammation in diabetic tissue repair.

## 3. Discussion

**Figure 6:**
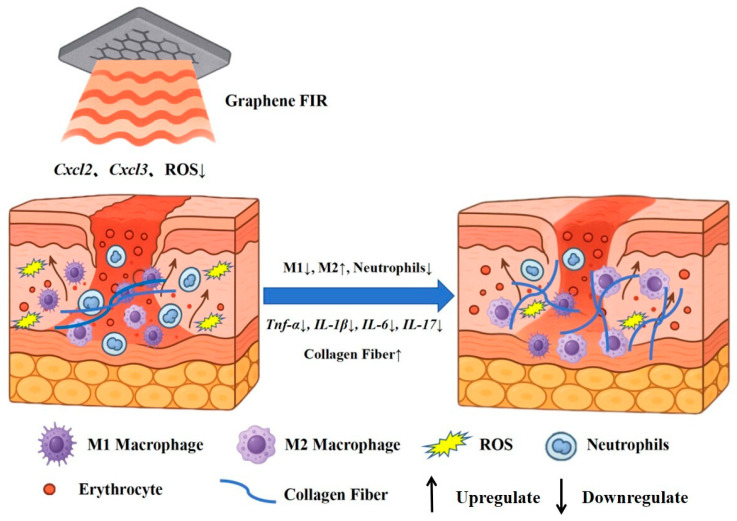
Proposed mechanism of graphene FIR therapy in promoting diabetic wound healing.

In this study, we provide compelling evidence that graphene-based far-infrared (FIR) therapy represents a non-invasive strategy to accelerate diabetic wound healing through coordinated modulation of inflammation and oxidative stress. As shown in Figure 6, graphene FIR promotes collagen fiber deposition, reduces ROS accumulation, and significantly downregulates pro-inflammatory chemokines Cxcl2 and Cxcl3. These effects collectively suppress neutrophil recruitment and M1 macrophage polarization, while promoting reparative M2 macrophages, culminating in markedly reduced levels of inflammatory cytokines (Tnf-α, IL-1β, IL-6, IL-17). The reduction in ROS levels was accompanied by decreased mRNA expression of the NF-κB subunits *p65* and *p50*, as well as their downstream inflammatory targets (Tnf-α and IL-1β). This concordance suggests that the antioxidant effects of FIR may contribute to suppression of the NF-κB–associated inflammatory network, thereby potentially interrupting the self-sustaining cycle of chronic inflammation. However, because NF-κB activation was not directly assessed, further studies examining phosphorylation status and/or nuclear translocation are warranted to confirm pathway engagement. Across these readouts, graphene FIR consistently outperformed MF, producing greater attenuation of oxidative stress and inflammatory signaling, together with more pronounced pro-regenerative responses. Such multi-target effects are consistent with reprogramming of the inflammatory microenvironment toward a healing-permissive state and promoting a more effective repair trajectory in diabetic wounds. To separate thermal contributions from material-specific effects, both GF and MF were applied under identical temperature control (41 ± 0.2 °C). Therefore, the superior performance of GF is unlikely to be explained solely by heating. This enhanced efficacy may be attributable to the distinct physical properties of graphene—particularly high thermal conductivity and strong far-infrared emissivity—which could enable more efficient and spatially uniform energy delivery, thereby establishing a more favorable local microenvironment than conventional metal-based heaters.

Beyond immunomodulation, the advantages of graphene FIR also stem from its intrinsic physical properties. Graphene’s high thermal conductivity and efficient far-infrared emission enable uniform, localized heating with minimal thermal loss. These features result in more consistent tissue penetration compared to metal films, which often exhibit uneven heat distribution and weaker FIR output. Biologically, this translated into more organized collagen deposition and enhanced angiogenesis in the graphene FIR group, as shown by trichrome and CD34 staining. While MF showed moderate improvements in tissue structure, it consistently lagged behind graphene FIR in both epidermal restoration and capillary density. Thus, graphene FIR uniquely integrates thermal precision with biological impact, offering clear advantages over conventional FIR platforms in promoting structural wound repair.

Moreover, graphene FIR demonstrated a superior capacity to suppress high-glucose-induced oxidative stress, a key barrier to healing in diabetic environments. In fibroblast models, graphene FIR significantly reduced intracellular ROS accumulation and attenuated downstream NF-κB-mediated cytokine expression, including Tnf-α and IL-1β. This antioxidative effect was consistently stronger than that observed with MF, which showed only partial suppression. The dual regulation of redox balance and immune signaling by graphene FIR suggests a comprehensive mechanism that addresses both causes and consequences of chronic inflammation. Unlike MF, which primarily serves as a passive heating source, graphene FIR actively modulates wound pathophysiology at multiple levels. This distinction underscores its potential as a next-generation FIR therapy, integrating material innovation with therapeutic precision to overcome the multifaceted barriers of diabetic wound healing.

Prior studies have shown that FIR therapy can exert anti-inflammatory and vasculoprotective effects via stress-response pathways, including induction of heme oxygenase-1, thereby attenuating cytokine-driven endothelial activation [64]. In preclinical models of diabetic wounds, FIR has also been reported to enhance granulation formation and neovascularization and to improve closure kinetics, supporting the notion that FIR can provide biological effects beyond passive warming when delivered under controlled conditions [65]. Consistent with this literature, the present work demonstrated that graphene-based FIR produced more robust immunomodulation and structural repair than a metal-film emitter. This advantage is likely attributable to graphene’s superior thermal properties, which may enable more uniform and localized energy delivery and mitigate uneven thermal fields that can compromise the comfort and safety of conventional thermal approaches [66].

From a translational perspective, hyperbaric oxygen therapy remains one of the best-established adjunctive interventions for refractory diabetic foot ulcers; however, its implementation is limited by chamber availability, time-intensive scheduling, and patient-specific contraindications [67,68]. Other oxygen-based approaches, including cyclic topical wound oxygen, may improve oxygenation but do not directly target inflammatory programming within the wound microenvironment [69]. Likewise, advanced dressing strategies such as sucrose octasulfate primarily modulate wound-bed biochemistry and do not provide a controllable physical stimulus [70]. In contrast, graphene FIR may offer a simpler and potentially more accessible alternative, as it can be designed for bedside or home use and readily integrated into wearable or dressing-like platforms.

Several limitations should be considered when interpreting these findings and defining subsequent translational steps. First, although the rat model and cell-based assays provided mechanistic insight, human diabetic wounds are more heterogeneous and are frequently influenced by ischemia, neuropathy, biofilm-associated infection, and repetitive mechanical trauma, all of which may alter thermal tolerance and the dominant inflammatory programs. Second, the exposure parameters were optimized for preclinical conditions; clinical translation will require device designs capable of delivering a reproducible thermal dose across anatomical sites and skin types while ensuring safety during prolonged or repeated application. Third, although suppression of NF-κB-linked inflammation and IL-17-associated cytokine signaling was observed within a 14-day window, longer-term immune adaptation, scar quality, and remodeling outcomes were not assessed. Fourth, protein-level validation was limited to immunohistochemistry for a restricted marker panel, which constrains pathway coverage and lacks the quantitative specificity of immunoblotting; accordingly, the absence of Western blot analyses limits inference regarding key signaling nodes and should be addressed in follow-up studies. Future work should therefore incorporate longer follow-up, include infected and ischemic wound models, and extend evaluation to larger animals with clinically relevant comorbidities. In addition, combination regimens that reflect real-world care—such as integration of graphene FIR with offloading, debridement, negative-pressure wound therapy, or evidence-based advanced dressings—should be tested to define settings in which additive benefit is most likely and to clarify the most practical clinical niche.

In conclusion, graphene FIR therapy enhanced diabetic wound healing through coordinated modulation of inflammatory chemokines, macrophage polarization, and oxidative stress. Its non-invasive property and broad immunoregulatory effects support clinical relevance for chronic wound management. Future studies should focus on validating graphene FIR across diverse wound models and on optimizing application regimens to ensure long-term safety and efficacy in human applications, thereby facilitating its integration into advanced wound care strategies.

## 4. Materials and Methods

### 4.1. Establishment of a Diabetic Wound Model

To establish a model of type 1 diabetes mellitus, diabetes was induced in rats by a single intraperitoneal injection of streptozotocin (STZ), which selectively destroys pancreatic β-cells, resulting in insulin deficiency and sustained hyperglycemia. Male *Sprague-Dawley* (SD) rats (7 weeks old, SPF grade) were acclimated for one week under standard laboratory conditions. Diabetes was induced by an intraperitoneal injection of streptozotocin (STZ) at a dose of 65 mg/kg, dissolved in a freshly prepared citrate buffer (pH 4.2–4.5, 10 mg/mL). All STZ preparations were protected from light and used within 30 min to ensure stability. Rats were fasted overnight (12 h, with water provided ad libitum) before injection and allowed to resume feeding two hours post-injection. To prevent initial hypoglycemia, a 5–10% sucrose solution was provided for 24 h post-injection before switching to regular water. Fasting blood glucose levels were measured from the tail vein on 48 and 72 h using a glucometer. Animals exhibiting blood glucose levels ≥16.7 mmol/L were monitored weekly, and those with consistent hyperglycemia (≥16.7 mmol/L for two consecutive weeks) were considered diabetic. For rats with glucose levels below the threshold, a supplementary injection of STZ (35 mg/kg) was administered. Once diabetes was confirmed, a full-thickness excisional wound model was created. Under isoflurane anesthesia, two 1.5 × 1.5 cm square wounds were generated on the dorsal skin using sterile surgical scissors, following disinfection and shaving of the skin. The entire skin layer, including the panniculus carnosus, was excised using forceps and scissors guided by a pre-marked sterile mold. Wounds were gently covered with sterile gauze to prevent contamination. A total of 32 rats were randomly assigned to four groups: Non-Diabetic Wound (NDW, n = 8), Diabetic Wound (DW, n = 8), Graphene Film-treated (GF, n = 8), and Metal Film-treated (MF, n = 8). Three rats were excluded from the final analysis due to wound induction failure (n = 2) and lethal anesthesia (n = 1). Thus, the final analyzed cohort consisted of 29 rats: Non-Diabetic Wound (NDW, n = 6), Diabetic Wound (DW, n = 7), Graphene Film-treated (GF, n = 8), and Metal Film-treated (MF, n = 8). The smaller control group (NDW) was statistically justified as it provided a common baseline for primary treatment comparisons, adhering to the ethical principle of reduction.

### 4.2. Local Graphene FIR Treatment

Prior to use, the graphene heating films were exposed to ultraviolet (UV) light for 30 min and disinfected with 75% ethanol. Following anesthesia, the graphene or metal film was applied directly to the wound surface and secured with medical adhesive tape. The surface temperature of the heating films was precisely maintained at 41 ± 0.2 °C using a temperature controller. Each rat received daily 45 min treatments for 14 consecutive days. During the treatment period, blood glucose levels and body weight were monitored regularly (Figure S2). Wound tissues were harvested on days 3, 7, and 14 after treatment for subsequent analyses and characterization.

### 4.3. Cell Culture and Treatment

RAW264.7 murine macrophages and human foreskin fibroblasts (HFFs) were cultured in Dulbecco’s Modified Eagle Medium (DMEM) supplemented with 10% fetal bovine serum (FBS) and 1% penicillin–streptomycin under standard conditions (37 °C, 5% CO_2_). To establish a high-glucose injury model, cells were incubated in DMEM containing 50 mmol/L glucose for 48 h prior to subsequent experiments. For M1 macrophage polarization, RAW264.7 cells were stimulated with 100 ng/mL lipopolysaccharide (LPS) and 20 ng/mL interferon-γ (IFN-γ) for 24 h. After polarization, cells were treated with either GF or a metal film MF placed on the culture plate surface. The films were connected to an alternating current (AC) power source, and the surface temperature was maintained at 41 ± 0.2 °C for 45 min. Cells were then harvested for downstream analyses.

### 4.4. Wound Tissue Collection

The study employed an independent cohort design. The tissue samples for molecular, histological, and immunological analyses were collected from separate, terminal cohorts of rats that were euthanized at each respective time point (days 3, 7, and 14). A total of 40 rats were randomly assigned to four groups (NDW, DW, GF, MF; n = 10 each). After excluding 4 rats due to wound induction failure, the final analysis included 36 rats, with 9 in each group (NDW, DW, GF, MF). Wound tissues were harvested on days 3, 7, and 14 after treatment. At each time point, 3 rats from each group were euthanized for sampling. Rats were anesthetized as described previously, and tissue samples were collected by excising the skin along the wound margins. A portion of the tissue was fixed in 4% paraformaldehyde for histological staining, while the remaining tissue was snap-frozen in liquid nitrogen and stored at −80 °C for subsequent molecular analyses.

### 4.5. Wound Area Measurement

Wound area was measured on days 3, 7, and 14 after treatment using image analysis. At each time point, rats were anesthetized, and the wound surface was gently cleaned with saline. Digital photographs of the wounds were taken at each time point with a reference scale placed adjacent to the wound, and the wound area was quantified using ImageJ software (National Institutes of Health, USA). Following scale calibration, the wound margin was manually traced with the polygon selection tool, and the enclosed area was calculated in mm^2^ by the ImageJ software. The wound healing rate (%) was calculated using the following formula:

Wound healing rate (%) = [(S_0_ − S_n_)/S_0_] × 100,

where S_0_ represents the initial wound area, and S_n_ denotes the remaining wound area at a given time point.

### 4.6. H&E Staining

Fresh wound tissues were fixed in 4% paraformaldehyde for 48 h, dehydrated through a graded ethanol series (75%, 85%, 95%, and absolute ethanol I and II), cleared with xylene I and II, and embedded in paraffin. Sections were cut at a thickness of 7 μm using a microtome, floated on a 45 °C water bath, mounted onto glass slides, and baked at 60 °C to remove moisture. For staining, sections were deparaffinized in xylene, rehydrated through a descending ethanol series (100%, 95%, 85%, 75%), and rinsed in distilled water. Slides were stained with hematoxylin for 5 min, washed in running tap water, differentiated briefly in 1% acid alcohol, and rinsed again. Eosin staining was then performed for 1 min, followed by dehydration, clearing in xylene, and mounting with neutral resin. Stained sections were examined under a light microscope.

### 4.7. Masson’s Trichrome Staining

Paraffin-embedded wound tissue sections were prepared as described for hematoxylin and eosin (H&E) staining. After deparaffinization and rehydration, slides were stained with freshly prepared Weigert’s iron hematoxylin (a 1:1 mixture of solutions A and B) for 5–10 min, followed by rinsing with distilled water. Sections were differentiated in acidic solution for 5–15 s, washed, and treated with Masson blue solution for 3–5 min to achieve bluing. After rinsing, sections were stained with Ponceau-Fuchsin solution for 5–10 min. A weak acid solution (prepared by mixing distilled water and weak acid at a 2:1 ratio) was used to rinse the slides for 30 s between staining steps. Sections were then treated with phosphomolybdic acid for 1–2 min, followed by aniline blue staining for 1–2 min. After a final rinse with the weak acid solution, sections were rapidly dehydrated using 95% ethanol and absolute ethanol, cleared in xylene, and mounted with neutral resin. Stained sections were air-dried at room temperature and examined under a light microscope.

### 4.8. CD34 Immunohistochemical Staining

Paraffin-embedded sections were dewaxed and rehydrated, then subjected to antigen retrieval in 0.01 mmol/L citrate buffer using microwave heating for 10 min, followed by cooling to room temperature. Sections were permeabilized with 0.5% Triton X-100, and endogenous peroxidase activity was blocked using 3% hydrogen peroxide. After blocking at room temperature for 1 h, sections were incubated overnight at 4 °C with anti-CD34 antibody (1:1000 dilution). The next day, HRP-conjugated secondary antibody was applied for 30 min, followed by DAB chromogenic development for approximately 2 min. Cell nuclei were counterstained with hematoxylin. Finally, slides were dehydrated, cleared in xylene, and mounted with neutral resin for microscopic examination.

### 4.9. Bulk RNA-Seq

RNA Extraction, Library Preparation, and Sequencing: Total RNA was extracted from rat skin using TRIzol reagent (Invitrogen, Carlsbad, CA, USA, Cat. No. 15596026) following the method by Chomczynski et al. [71]. DNA digestion was performed using DNase I after RNA extraction. RNA purity was assessed by measuring the A260/A280 ratio using a NanoDrop™ OneC spectrophotometer (Thermo Fisher Scientific Inc., Waltham, MA, USA), and RNA integrity was confirmed via 1.5% agarose gel electrophoresis. RNA quantity was determined using a Qubit™ 3.0 fluorometer with the Qubit™ RNA Broad Range Assay Kit (Life Technologies, Carlsbad, CA, USA, Q10210). Two micrograms of total RNA were used for stranded RNA sequencing library preparation using the Ribo-off rRNA Depletion Kit (Human/Mouse/Rat) (Cat. No. MRZG12324, Illumina, San Diego, CA, USA) and the KC-Digital™ Stranded mRNA Library Prep Kit for Illumina^®^ (Cat. No. DR08502, Wuhan Seqhealth Co., Ltd., Wuhan, China), following the manufacturer’s instructions. This kit reduces PCR and sequencing duplication bias by using an 8-base unique molecular identifier (UMI) to label pre-amplified cDNA molecules. Library products of 200–500 base pairs were enriched, quantified, and sequenced on a DNBSEQ-T7 sequencer (MGI Tech Co., Ltd., Shenzhen, China) using the PE150 mode.

RNA-Seq Data Analysis: Raw sequencing data were first filtered using Trimmomatic (v0.36) to remove low-quality reads and adapter contamination. Clean reads were further processed using custom in-house scripts to eliminate duplication bias introduced during library preparation and sequencing. In brief, reads were clustered by UMI sequences, and those with identical UMIs were grouped into clusters. Within each cluster, pairwise alignment was used to identify reads with over 95% sequence identity, which were then grouped into sub-clusters. A multiple sequence alignment was performed on each sub-cluster to generate a consensus sequence, thereby removing PCR and sequencing errors. Reads mapping to exon regions were counted using featureCounts (Subread v1.5.1, Bioconductor), and expression levels were calculated as RPKM. Differentially expressed genes (DEGs) between groups were identified using the edgeR package (v3.12.1) with a *p*-value cutoff of 0.05 and a fold-change threshold of 2. Gene Ontology and Kyoto Encyclopedia of Genes and Genomes (KEGG) enrichment analyses of DEGs were performed using KOBAS (v2.1.1) with a *p*-value cutoff of 0.05 to determine statistical significance. Alternative splicing events were detected using rMATS (v3.2.5) with a false discovery rate (FDR) cutoff of 0.05 and an absolute value of Δψ ≥ 0.05.

### 4.10. Immunofluorescence Staining

Paraffin-embedded tissue sections were dewaxed and rehydrated through xylene and a graded ethanol series, followed by washing in distilled water. Antigen retrieval was performed by heating the sections in a citrate-based buffer using three microwave cycles (3 × 3 min), followed by cooling and rinsing under running water. Endogenous peroxidase activity and non-specific binding were blocked using commercial blocking solutions, each incubated for 10 min at room temperature. Primary antibodies (1:800 dilution) were applied and incubated overnight at 4 °C. The next day, slides were brought to room temperature, washed with phosphate-buffered saline (PBS; 3 × 5 min), and incubated with HRP-conjugated secondary antibodies for 30 min. Fluorescent dye-conjugated reagents were then applied for 5 min to visualize target proteins, followed by additional PBS rinses. Nuclei were counterstained with DAPI for 5 min in the dark, washed with PBS, and mounted using an anti-fade mounting medium. Slides were then examined under a fluorescence microscope.

### 4.11. Enzyme-Linked Immunosorbent Assay (ELISA)

Wound tissues were homogenized in 1× phosphate-buffered saline (PBS) at a 1:100 (*w*/*v*) ratio, centrifuged at 5000 rpm for 5 min, and the supernatant was collected. Tnf-α and IL-6 capture antibodies (1:1000 dilution) were coated onto 96-well plates and incubated overnight at 4 °C. After washing, the wells were blocked with 5% skim milk for 2 h at room temperature. Diluted tissue samples were then added and incubated for 2 h. Horseradish peroxidase (HRP)-conjugated secondary antibodies were applied and incubated for 1 h in the dark, followed by color development and reaction termination using sulfuric acid (H_2_SO_4_). Absorbance was measured at 450 nm, and cytokine concentrations were determined using a standard curve.

### 4.12. Quantitative Real-Time PCR (qRT-PCR)

Total RNA was extracted from wound tissues or cultured cells using TRIzol reagent, and reverse transcription was performed using a commercial cDNA synthesis kit according to the manufacturer’s instructions. qRT-PCR was conducted using SYBR Green Master Mix on a real-time PCR system. Relative gene expression levels were calculated using the 2^−ΔΔCt^ method, with *GAPDH* used as the internal reference gene. Primer sequences are provided in Tables S1 and S2.

### 4.13. Statistics

Data were expressed as mean ± SEM. Statistical comparisons between two groups were performed using Student’s two-tailed *t*-test. When the data failed the normality test, the non-parametric Mann–Whitney *U*-test was used instead. For comparisons involving more than two groups, a one-way ANOVA was conducted, followed by Tukey’s or Bonferroni’s tests. A *p*-value of less than 0.05 was considered statistically significant. All statistical analyses were carried out using the Statistical Package for the Social Sciences (SPSS 29.0). Significance levels were denoted as follows: * *p* < 0.05, ** *p* < 0.01, *** *p* < 0.001, **** *p* < 0.0001.

## Data Availability

All data are available in the main text or the Supplementary Materials.

## Supplementary Materials

The following supporting information can be downloaded at: https://www.mdpi.com/article/10.3390/ijms27073101/s1.

## Abbreviations

FIR	far-infrared
MF	metal film
GF	graphene film
DFUs	diabetic foot ulcers
ROS	reactive oxygen species
DW	diabetic wound
NDW	non-diabetic wound
GO	gene ontology
KEGG	Kyoto Encyclopedia of Genes and Genomes
LPS	lipopolysaccharide
IFN-γ	interferon-γ
STZ	streptozotocin
UV	ultraviolet
HFFs	human foreskin fibroblasts
