# Supplementary material for: Graphene Far-Infrared Therapy Enhances Diabetic Wound Healing Through Potential Mitigation of Oxidative Stress and Inflammation and Regulation of Chemokines and Macrophage Polarization
Source: Int J Mol Sci. 2026 Mar 29;27(7):3101. doi: 10.3390/ijms27073101 (PMC13073909; doi:10.3390/ijms27073101)
Supplement: Supplementary file 1 [file ijms-27-03101-s001.zip › ijms-4094279-supplementary.pdf]

Supplementary Materials

Graphene Far-Infrared Therapy Enhances Diabetic Wound Healing Through Potential Mitigation of Oxidative Stress and Inflammation and Regulation of Chemokines and Macrophage Polarization

Xinyu Jian †, Xuanjun Wu †, Xian Luo ‡, Chengwei Cao ‡, Qianwen Wu, Ziwen Chen, Zhichao Hu, Hua Zhu and Binghui Wu *

Innovation Laboratory for Sciences and Technologies of Energy Materials of Fujian Province (IKKEM), State Key Laboratory for Physical Chemistry of Solid Surfaces, Collaborative Innovation Center of Chemistry for Energy Materials (iChEM), National and Local Joint Engineering Research Center of Preparation Technology of Nanomaterials, College of Chemistry and Chemical Engineering, Pen-Tung Sah Institute of Micro-Nano Science and Technology, College of Medicine, Xiamen University, Xiamen 361005, China; jianxinyu2001@stu.xmu.edu.cn (X.J.); 36520211151875@stu.xmu.edu.cn (X.W.); 24520220157307@stu.xmu.edu.cn (X.L.); 17720880695@163.com (C.C.); 36520221151880@stu.xmu.edu.cn (Q.W.); 36520211151718@stu.xmu.edu.cn (Z.C.); hu13545590469@163.com (Z.H.); zh_980903@163.com (H.Z.)

* Correspondence: binghuiwu@xmu.edu.cn

† These authors contributed equally to this work.

‡ These authors also contributed equally to this work.

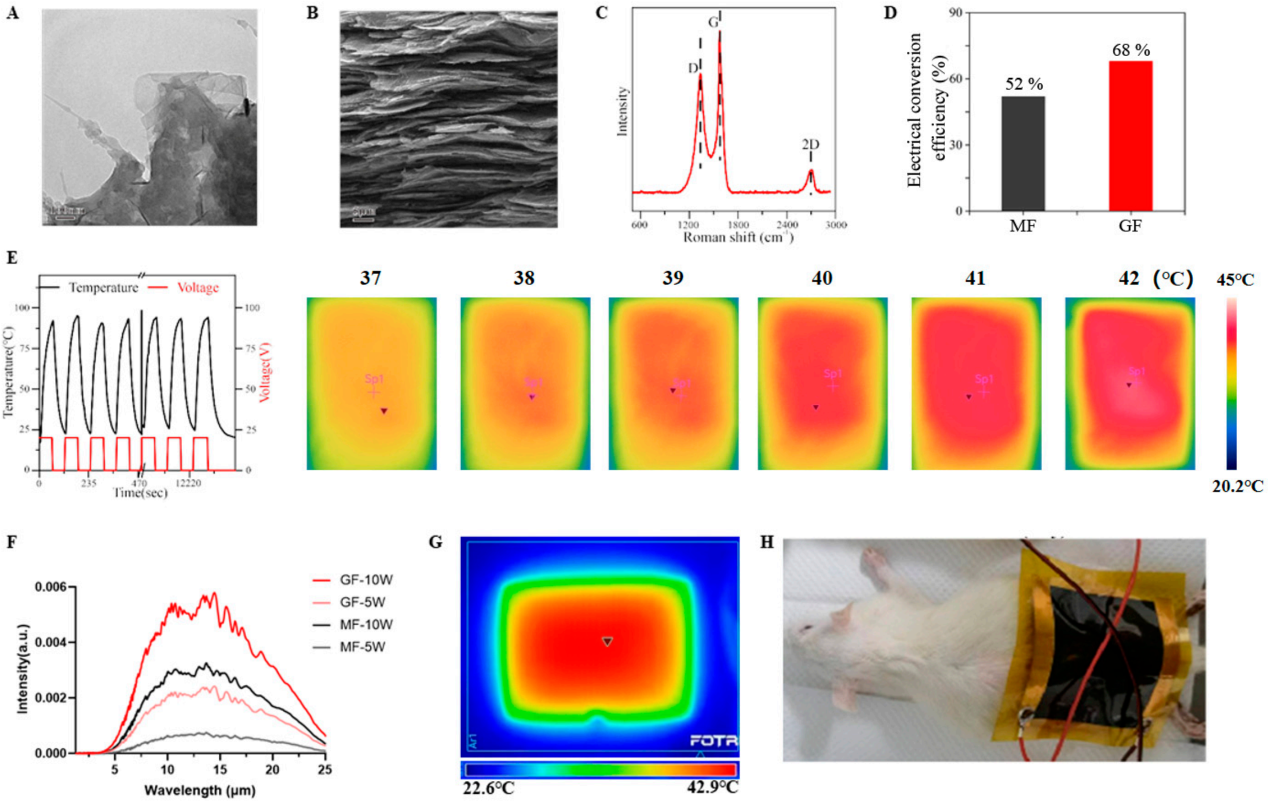

Figure S1. (**A**,**B**) TEM and SEM images of materials derived from graphene ink. (**C**) Raman characterization of graphene films. The D peak originates from carbon black nanoparticles, while the G and 2D peaks are attributed to single-layer graphene and multilayers with fewer than 10 layers. (**D**) Electrical conversion efficiency of MF and GF. (**E**) Temperature curve of graphene films subjected to a square wave voltage ranging from 0 to 20 V. The infrared thermal images of GF with different temperatures. (**F**) Far-infrared emission spectra of GF and MF at different power levels. (**G**) Thermal

imaging of a far-infrared emitting device under a power of 4.5 W. (**H**) Schematic diagram of treatment for diabetic wounds in rats.

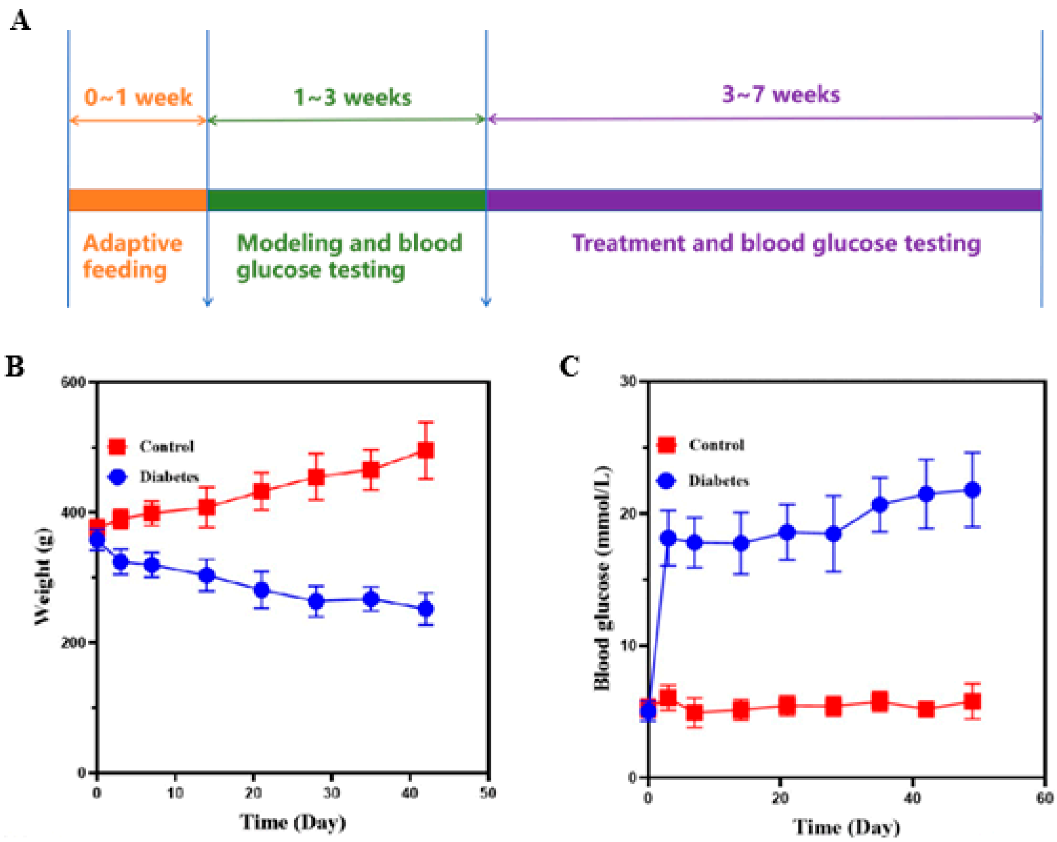

Figure S2. (**A**) Timeline of the animal experiment. (**B**) Changes in fasting body weight and (**C**) fasting blood glucose of rats following STZ injection.

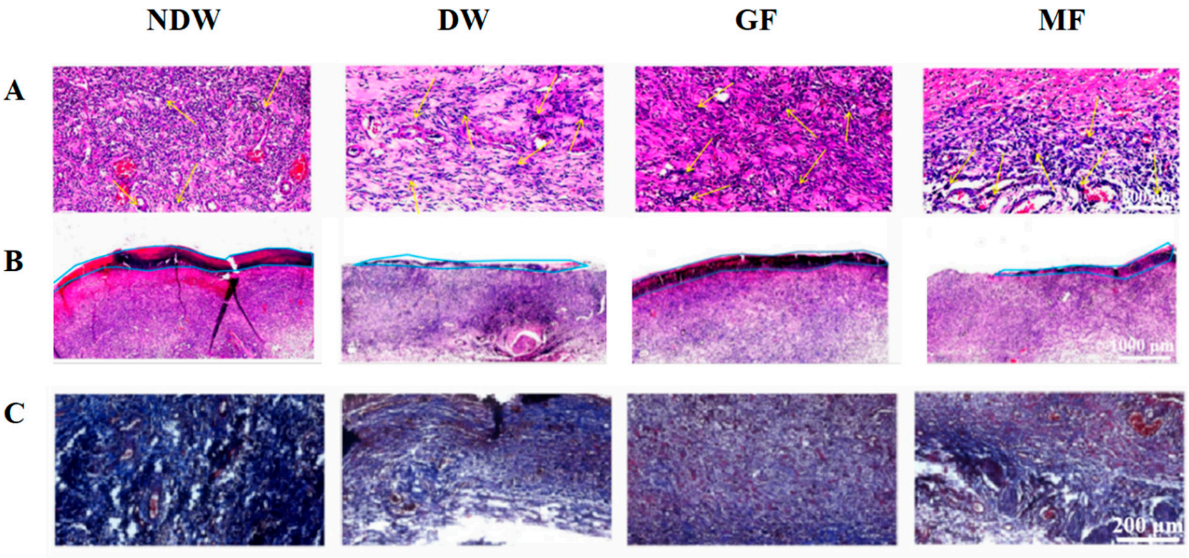

Figure S3. (**A**) Hematoxylin and eosin (HE) staining results of rat skin 3 days after treatment. (**B**) Healing outcomes of the epidermal layer in rats 7 days after treatment. (**C**) Collagen fiber deposition results in rats 3 days after treatment.

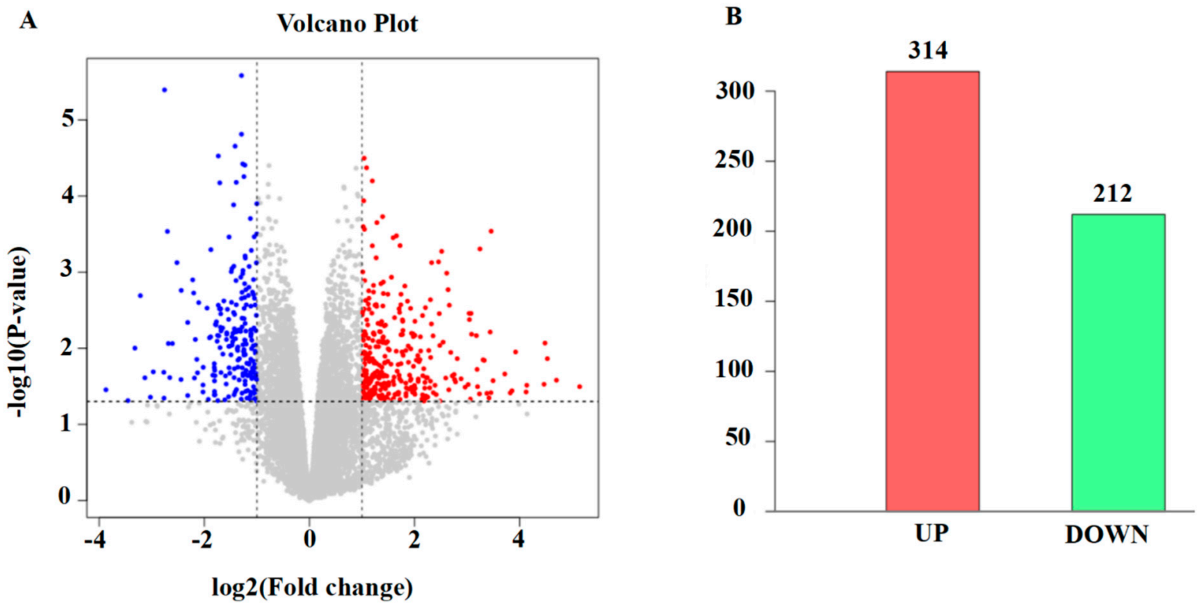

Figure S4. Bulk RNA-Seq sequencing results. (**A**) Volcano plot of differentially expressed genes between the GF and DW groups 7 days after treatment. (**B**) Statistical results of differentially expressed genes between the GF and DW groups: compared with the DW group after GF treatment, 314 genes were upregulated and 212 genes were downregulated.

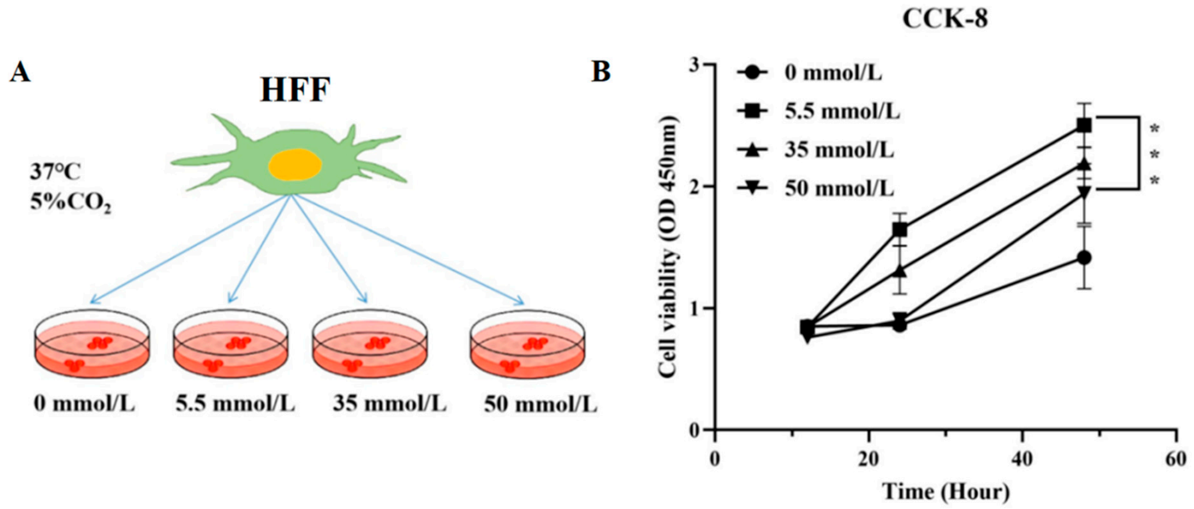

Figure S5. (**A**) Schematic diagram of the high-glucose-induced injury model in human foreskin fibroblast (HFF). (**B**) Results of the high-glucose-induced injury model in cells.

Table S1. PCR primer sequences for animal experiments.

Gene	Primer
Cxcl2	F：TCCCTCCTGTGCTCAAGACT<br>R：ACCCCTGTACCCTGATGGTT
Cxcl3	F：GCTGGGGTAGTCAGGAAACA<br>R：TCGATCATTCCTGTGACGCT
Tnf-α	F：CTTCTCATTCCTGCTCGTGG<br>R：TGATCTGAGTGTGAGGGTCTG
IL-1β	F：TGCAGGCTTCGAGATGAAC<br>R：GGGATTTTGTCGTTGCTTGTC
IL-6	F：AAGCCAGAGTCATTCAGAGC<br>R：GTCCTTAGCCACTCCTTCTG

IL-17	F：GTTCAGTGTGTCCAAACGCC<br>R：AGGGTGAAGTGGAACGGTTG
P65	F：CTACGAGACCTTCAAGAGCATC<br>R：GATGTTGAAAAGGCATAGGGC
P50	F：AGACGACGATCCTTTCGGAAC<br>R：AAGGTATGGGCCATCTGTTGA
β-actin	F：CACTCCAAGTATCCACGGCA<br>R：TCCTCCCCTTAGGAGTTGGG

Table S2. PCR primer sequences for cell experiments.

Gene	Primer
Tnf-α	F：GATCGGTCCCCAAAGGGATG<br>R：CCACTTGGTGGTTTGTGAGTG
IL-1β	F：TGCCACCTTTTGACAGTGATG<br>R：TGATGTGCTGCTGCGAGATT
IL-6	F：GACAAAGCCAGAGTCCTTCAGA<br>R：TGTGACTCCAGCTTATCTCTTGG
CD86	F：CTGTATAAGGACGCCCAGGAG<br>R：ACAGCAGCATTCCCGAAGAT
IL-10	F：AGGCGCTGTCATCGATTTCT<br>R：ATGGCCTTGTAGACACCTTGG
Fizz1	F：ATGACTGCTACTGGGTGTGC<br>R：CAGTGGTCCAGTCAACGAGT
Chil3	F：GGGCCCTTATTGAGAGGAGC<br>R：GCACTGTGGAAAAACCGTTGA
CD206	F：GAAGAGGTGGAGAACCCAGC<br>R：AGAAGCAGGGTAAATGGGCA
GAPDH	F：CCCTTAAGAGGGATGCTGCC<br>R：TACGGCCAAATCCGTTCACA
Gclc	F: ACACCTGGATGATGCCAACGAG<br>R: CCTCCATTGGTCGGAACTCTAC
Gpx1	F: CGCTCTTTACCTTCCTGCGGAA<br>R: AGTTCCAGGCAATGTCGTTGCG
Cat	F：CGGCACATGAATGGCTATGGATC<br>R：AAGCCTTCCTGCCTCTCCAACA
Sod1	F：GGTGAACCAGTTGTGTTGTCAGG<br>R：ATGAGGTCCTGCACTGGTACAG
Sod2	F：TAACGCGCAGATCATGCAGCTG<br>R：AGGCTGAAGAGCGACCTGAGTT
Nrf2	F：CAGCATAGAGCAGGACATGGAG<br>R：GAACAGCGGTAGTATCAGCCAG
